# The effects of advanced factor analysis approaches on outcomes in randomised trials for depression: protocol for secondary analysis of individual participant data

**DOI:** 10.1192/bjo.2023.544

**Published:** 2023-08-11

**Authors:** Frank Doyle, David Byrne, Robert M. Carney, Pim Cuijpers, Alexandra L. Dima, Kenneth Freedland, Suzanne Guerin, David Hevey, Bishember Kathuria, Shane Kelly, Stephen McBride, Emma Wallace, Fiona Boland

**Affiliations:** Division of Population Health Sciences, RCSI University of Medicine and Health Sciences, Ireland; Department of Psychiatry, Washington University School of Medicine, Missouri, USA; Department of Clinical, Neuro and Developmental Psychology, Vrije Universiteit Amsterdam, The Netherlands; Health Psychology and Health Services, Sant Joan de Déu Research Institute, Spain; School of Psychology, University College Dublin, Ireland; School of Psychology, Trinity College Dublin, Ireland; Digital Transformation, Novartis Ireland Ltd, Dublin, Ireland; Psychological Society of Ireland, Dublin, Ireland; Aware, Dublin, Ireland; Department of General Practice, University College Cork, Ireland; and Department of General Practice, RCSI University of Medicine and Health Sciences, Ireland

**Keywords:** Major depressive disorder, adult psychiatry, mental health, randomised trial, psychometric

## Abstract

**Background:**

Modern psychometric methods make it possible to eliminate nonperforming items and reduce measurement error. Application of these methods to existing outcome measures can reduce variability in scores, and may increase treatment effect sizes in depression treatment trials.

**Aims:**

We aim to determine whether using confirmatory factor analysis techniques can provide better estimates of the true effects of treatments, by conducting secondary analyses of individual patient data from randomised trials of antidepressant therapies.

**Method:**

We will access individual patient data from antidepressant treatment trials through Clinicalstudydatarequest.com and Vivli.org, specifically targeting studies that used the Hamilton Rating Scale for Depression (HRSD) as the outcome measure. Exploratory and confirmatory factor analytic approaches will be used to determine pre-treatment (baseline) and post-treatment models of depression, in terms of the number of factors and weighted scores of each item. Differences in the derived factor scores between baseline and outcome measurements will yield an effect size for factor-informed depression change. The difference between the factor-informed effect size and each original trial effect size, calculated with total HRSD-17 scores, will be determined, and the differences modelled with meta-analytic approaches. Risk differences for proportions of patients who achieved remission will also be evaluated. Furthermore, measurement invariance methods will be used to assess potential gender differences.

**Conclusions:**

Our approach will determine whether adopting advanced psychometric analyses can improve precision and better estimate effect sizes in antidepressant treatment trials. The proposed methods could have implications for future trials and other types of studies that use patient-reported outcome measures.

## Psychometric assessment

Multi-item psychometric scales are ubiquitous in social, behavioural and clinical sciences.^1,2^ Such scales are commonly used to assess individual and group differences in areas such as attitudes, behaviours and mood, and can be self-reported or observer-rated. These scales are so important that entire subfields of research largely depend on them (e.g. clinical and health psychology), and they are typically used both as predictors and outcomes. Ongoing instrument validation, including using statistical psychometric analysis, is recommended.^1,2^ This is necessary because validity is not inherent in these instruments, but may instead be tied to the sample analysed (i.e. in classical test theory) or to other contextual factors. Over several decades, sophisticated theories and statistical methods have been used to ascertain psychometric validity, such as classical test theory (e.g. exploratory and confirmatory factor analysis), item response theory (both parametric and non-parametric methods, e.g. Rasch modelling, three-parameter logistic modelling, Mokken scaling, etc.)^1–3^ and, more recently, network analysis.^4,5^ Network analysis departs from the usual methods of establishing uni- or multi-dimensionality, to instead estimate complex patterns of inter-relationships among different scale items. User-friendly software for performing these types of analyses is now available, making modern psychometric methods accessible to a wider range of researchers.

However, despite psychometricians’ contention that ongoing scale validation is necessary, it is unclear to what extent such analyses add value to the studies that use these instruments. There are several reasons why these processes may not be convincing and should be subject to a cost–benefit analysis. Different techniques can yield different, often conflicting, results for the same scales;^4–6^ large sample sizes are required for appropriate analyses^2,7^ and there is potential for never-ending cycles of ongoing validation. In addition, psychometric expertise may be lacking in those who most commonly use the instruments, and resource costs for needed expertise and training should be considered. However, perhaps most concerning of all is that when complex analyses are completed, it may be unclear as to whether anything important has been learned. For example, Dima^3^ recently proposed a very comprehensive, six-step protocol for psychometric assessment, covering the main areas of classical test theory and item response theory. To illustrate these processes, they analysed data from 222 people with chronic pain who had completed the 24-item Sickness Impact Profile – Roland Scale. The analyses yielded a unidimensional scale of 15 items instead of the 24 original items, which is a useful reduction in participant burden. However, the final scale correlated at 0.97 with the original scale, suggesting that subsequent outcome analyses would not be substantially affected by using the shorter, 15-item version. In contrast, there are benefits to such analysis, such as reduced respondent burden with probable better completion of measures, scientific and theory development, and potentially reduced measurement error, which is a strength particularly of confirmatory factor analytic approaches.^1–3^ However, sample sizes of individual trials may not be sufficient to conduct such analyses, even if the potential benefits are valued.

## When a treatment matters: estimating effects in randomised trials

The effects or efficacy of any given treatment or intervention can be determined by randomised trials. Thus, if we want to contend that psychotherapy or antidepressants are helpful in treating depression, randomised trials are used to determine the efficacy of treatment relative to a comparator group. Cuijpers et al^8^ recently published a network meta-analysis of 101 studies of 11 910 people with moderate-to-severe depression, which showed that a combination psychotherapy and antidepressants is more effective than either one alone. Similarly, Cipriani et al, in a network meta-analysis of antidepressant treatments based on 522 trials and 116 477 participants, showed an overall positive treatment effect for antidepressants.^9^ These types of meta-scientific approaches allow for estimation of treatment effects that cannot be achieved by single studies. In addition, there are potentially important subgroup effects, such as well-established gender differences in the prevalence of depression,^10–12^ although gender may not be important for treatment effects (e.g. mean differences were smaller than one point on the Hamilton Rating Scale for Depression (HRSD) at post-test in one review).^13^ Although this suggests that the efficacy of psychotherapies and pharmacotherapies do not vary by gender, it is not known whether gender affects psychometric analysis of depression trials in terms of measurement invariance of clinically important differences among particular depression items.

## Could psychometrics really matter in trials? The current proposal

It is unclear whether psychometric analysis ‘matters’, in terms of the results of primary outcome analysis or for subgroup analyses. We therefore propose to combine individual patient data (IPD) from depression treatment trials to obtain sufficient sample sizes to apply advanced psychometric approaches, and to determine whether these analyses make a difference to the effects of depression treatment studies. Specific objectives are: (a) to establish psychometric models of depression pre- (baseline) and post-treatment (outcome), using the 17-item version of the HRSD (HRSD-17), yielding one or more factors of depression; (b) to determine overall psychometrically informed depression effect size according to treatment group, and compare with the original treatment effect size; (c) to compare psychometrically informed effect sizes and original effects across trials and (d) to compare proportions achieving remission using risk differences according to the recommended thresholds for the HRSD-17 versus the equivalents for the new psychometrically informed data across trials.

## Method

### Study design

We will conduct a secondary analysis of IPD aggregated across multiple randomised antidepressant treatment trials. Data requests have been submitted to Clinicalstudydatarequest.com (CSDR) and Vivli.org, which are international databases of clinical trials, to obtain pharmacotherapy trial data that fit the inclusion criteria outlined in Table 1 below. Although CSDR hosts trials from several sponsors, it emerged that all 65 trials that met the inclusion criteria and had data/supporting documentation available were sponsored by GlaxoSmithKline (GSK). Of the 42 studies requested from Vivli.org, 13 were sponsored by Eli Lilly and Company, eight were sponsored by GSK, nine by Takeda and 12 by Pfizer. IPD from these studies will be pooled according to the database from which they were obtained, and subject to separate psychometric analyses. Randomised controlled trials for depression typically adopt versions of the HRSD^14^ as outcome measures. We will use the 17 items that are common to the HRSD-17 in all instances, to maximise the sample size. A comparison of effect sizes obtained from the original trial data (which have not been informed by psychometrics) and the factor-informed effects will be assessed. A threshold at which standardised mean differences (SMDs) will be considered clinically important will be established as per Cuijpers et al,^15^ who tentatively proposed a threshold of SMD = 0.24. The results of effect size comparisons will be interpreted to determine whether using such psychometric techniques leads to different results, and whether the differences exceed this threshold, i.e. whether they are large enough to be considered clinically important. Potential moderators, such as gender, study country and type of antidepressant, will also be investigated. The open-source software packages R version 4.3.1 for Windows (The R Foundation for Statistical Computing; see https://www.r-project.org/), SAS version 9.4 for Windows (SAS, Cary, North Carolina, USA: see https://support.sas.com/software/94/)^16^ and Stata version 17 for Windows (StataCorp, College Station, Texas, USA; see https://www.stata.com/) will be used for the statistical analyses.

**Table 1:** Trial/participant selection criteria

Variable	Criteria
Phase	2, 3 and 4 randomised controlled trial
Population	Participants with any depressive disorder aged 18 years and older who were included in antidepressant treatment trials
Intervention	Antidepressant medication (any used in the context of treating depression)
Comparator	Any comparator (to establish baseline psychometric model); placebo (to establish efficacy)
Timing of outcome Assessment	8 weeks (or within a band of 4–12 weeks), as per Cipriani et al^9^ and Doyle et al^17^
Exclusion criteria	All studies outside of the above parameters

### Participants

The study is open to the inclusion of data from any randomised controlled trial that has examined the effects of pharmacotherapy, in relation to any comparator (to establish the baseline psychometric model), or to placebo only (to establish the efficacy effects). The specific data composition required for inclusion in this study is outlined in Table 1.

Individual trial sample size is not an eligibility criterion in our study. All data will be pooled for psychometric modelling. Preliminary data checks suggest that approximately 65 studies from CSDR and 45 studies from Vivli.org can be obtained that meet the above criteria, providing a total possible sample of approximately 10 000 participants for each database. Numbers will be reduced when confining the data to the trials that use item-level HRSD data or some variant thereof, or on careful checking of the remaining inclusion criteria, but we expect a sample size in excess of 5000 participants each for both CSDR and Vivli.org databases.

### Outcome assessment: HRSD

The HRSD^14^ is among the most widely used clinician-administered assessment instrument in randomised trials for depression. Originally conceived as a 17-item composite scale, the HRSD is currently available in versions ranging from seven to 31 items in length, and has also been published in several different languages. This study will garner IPD from the 17 items that are common to the most frequently used version, to maximise sample size (HRSD-17). These 17 items are specifically designed to measure melancholic and physical symptoms of depression, and vary in their scale of measurement, with seven items measured on a three-point scale (usually from 0 to 2) and ten items measured on a five-point scale (usually from 0 to 4).

Although it has long been considered the ‘gold standard’ for the assessment of depression in randomised trials, the HRSD has come under scrutiny in recent years. Particularly, poor replication of the factor loading structure has been noted in previous research, which may result in nonperforming items being omitted in optimal psychometric models.^18^ Collinearity may also be present among similar items, such as the three ‘insomnia items’ or the three ‘somatic’ items, which could violate the assumptions of factor analysis or cause computation failures in factor analyses. In these cases, we will assess the creation of composite ‘sum score’ items and their effect on model fit. Therefore, psychometric analyses could result in several nonperforming items being dropped from the model.

For the primary outcome analysis, scores will be modelled as continuous variables. However, to model the results as thresholds requires a different approach to maintain equivalence. The current recommended threshold (≤7) as a percentage of the maximum possible depression score for the 17-item model (i.e. 52) is 13%. We will therefore calculate the revised threshold as 13% of the maximum possible score of the factor-informed model of the HRSD. For example, if analysis indicates a 12-item model with a maximum possible score of 39, 13% of this score would result in a depression threshold of ≤5 for the psychometrically informed model to achieve remission. Thus, 13% of the maximum score will be used to indicate remission for our secondary outcome analysis (detailed below).

### Statistical analysis plan

We will first examine psychometric models of depression pre- (baseline) and post-treatment (outcome), measured using the HRSD-17, to obtain one or more factors. We will also examine a combined outcome model, and the factor structure of the optimum baseline model and optimum combined outcome model of depression will then be used to compute factor scores. Multilevel models incorporating a random effect for trial will be used to ascertain factor-informed change scores. Subsequently, for each individual trial data-set, factor-informed change scores will be ascertained and compared against the change scores of the original raw trial data, to identify statistical and clinically important differences in trial outcomes. Differences in individual trial effects (original and modified) will then be meta-analysed to obtain a pooled effect size of change between the original and modified data. These methods are described below (for a detailed statistical analysis plan and schematics of each analysis, see Supplementary Material available at https://doi.org/10.1192/bjo.2023.544). The complete psychometric and effect size analyses will be conducted with Vivli.org and CSDR data separately, with any differences in depression models or effect size outcomes reported. All analyses conducted and any differences obtained using the two repositories will be reported. Results will not be reconciled using statistical approaches. Instead, any inconsistencies, if they exist, and the potential implications (e.g. test instrument may be unstable, or particular items may perform differentially between the data-sets) will be reported in full and discussed.

### Psychometric analysis

#### Establish psychometric models of depression pre- (baseline) and post-treatment (outcome), using the HRSD-17

Factor models will be ascertained for combined pre-treatment (baseline) and separately for intervention and placebo outcome groups. In line with the approach recommended by Lubke and Lunningham,^19^ data will be split randomly into two groups; an ‘exploratory data-set’ and a ‘confirmatory data-set’. We will undertake to perform this split in relation to each individual trial and within treatment group, to ensure an equitable representation of each trial in the exploratory and confirmatory data-sets. Using the exploratory data-set, parallel analysis will be applied to the HRSD-17 to determine the dimensionality of the data,^20^ and exploratory factor analysis will be conducted to determine the factor structure for baseline, individual placebo and treatment outcome groups, and a combined outcome group.^20^ Mokken^5,21^ scaling approaches will be considered if results are ambiguous. Once exploratory factor analysis has determined the dimensionality and factor structure of the data, confirmatory factor analysis^22^ will be conducted with the confirmatory data-set in relation to the simple structure exploratory factor analysis model, along with two common factor analytical models that are well defined and replicated in psychopathology research: a higher-order model and a hierarchical bi-factor model.^23^ The optimum model of depression will be identified by using a number of model selection criteria and fit indices, as well as chi-squared tests of the factor analytical models, according to established practices,^22,24^ and this model will be applied to the combined total sample to calculate factor scores for later analyses. Model selection criteria will include Akaike information criterion and Bayesian information criterion. Absolute fit indices will include root mean square error of approximation and standardised root mean square residual, which are considered acceptable at <0.08.^25^ Relative fit indices will include comparative fit index and Tucker–Lewis index, which are considered acceptable at >0.95.^26^ Multivariate normality of the data will be examined with the Henze–Zirkler test.^22^ This will determine whether a robust maximum likelihood method will be required when estimating model parameters.

Potential gender effects will be examined by using measurement invariance methods. Weak invariance will be examined by constraining factor loadings across gender groups, whereas strong invariance will be examined by constraining factor loadings and intercepts.^24^ The presence or absence of gender invariance will then be established by using the lavTestLRT function in lavaan (version 0.6-9),^27^ to compare the goodness of fit of the initial and constrained models. Statistically significant differences will be examined for differences that would be considered clinically significant. In this regard, differential item functioning will be assessed by examining a range of statistics, such as factor loading scores, *r*^2^ values, mean values, s.d. and s.e. If gender variance is found, individual psychometrically optimised models will be examined by using data for men and women separately.^22,24^

The models produced at baseline and outcome will be examined to identify any potential differences in model structure or item functioning. This will include examination of differences in nonperforming (i.e. dropped) items, examination of the factors onto which each item loads and inspection of the salience of each item according to their respective factor loading scores. Factor score weights will be garnered from the baseline model and the combined outcome model that performs best, and used to obtain factor scores for each trial for use in later analyses. A schematic of the planned psychometric analyses is available in the Supplementary Material.

#### Modelling change and calculating effect sizes

##### Comparing changes in depression factors to changes in original HRSD-17 scores: continuous outcomes (primary outcomes)

A sequence of analyses will be performed to establish whether there is any evidence that the application of factor scores modifies the obtained effect sizes. First, we will establish the overall effects of antidepressants versus placebo in the pooled data, using original (raw score) HRSD-17 scores, by using a multilevel linear regression model, predicting outcome HRSD-17 scores, with treatment group as the predictor, adjusting for baseline HRSD-17 scores and with study as the random intercept (model 1). Then, a similar model will be built, except the total factor score at outcome (i.e. psychometrically informed outcome sum scores) will be modelled, adjusting for total factor score at baseline (model 2). We will also re-run these two models (i.e. models 1 and 2), additionally adjusting for participant age and gender (models 3 and 4, respectively). In each case, effects will be transformed into Cohen's *d* SMD to allow for effect size comparison.

Furthermore, each trial will be examined individually, to assess changes per trial, and results combined in a meta-analysis. Linear regression analysis will be used separately for the original trial data and factor score data, to assess potential changes in outcome scores adjusting for baseline scores. Effect sizes will be obtained for each trial, comparing the original effect sizes from the HRSD-17 total scores and the factor-informed scores, again calculating SMDs. The difference between the (raw score) SMD and the factor-informed SMD is considered the effect of interest for each trial.

Differences in effect sizes from the original and factor score results will be calculated and assessed by using random-effects meta-analysis^28^ to determine an overall change in effect size for the psychometrically optimised data. We will also examine potential moderators of the found effects, including study country, antidepressant type and gender, in a meta-regression analysis. A schematic of the planned primary analyses is available in the Supplementary Material.

##### Comparing proportions achieving remission (secondary outcome)

Clinically important differences between original and psychometrically informed data will also be assessed by examining for potential changes in the number and percentage of participants who achieve remission, as well as differences in absolute risk reduction. These analyses will be conducted with the recommended^14^ remission threshold of ≤7, and then repeated by using the psychometrically determined threshold mentioned above (i.e. 13% of the factor score). Remission rates and absolute risk differences will then be examined, to identify potential differences in the number of participants who achieve remission according to the original and psychometrically informed data.

Similar to the primary analysis, overall multilevel models will be built, this time with logistic regression analysing the participants who achieve remission, using the raw data and the psychometrically informed data. Further models will also adjust for age and gender.

Then, we will use similar procedures to obtain the numbers achieving remission per trial, which will ultimately be modelled by using random-effects meta-analysis, with meta-regression exploring the potential moderators. The parameters for the meta-analyses are outlined in Table 2. A schematic of the planned secondary analyses is available in the Supplementary Material.

**Table 2:** Parameters for meta-analyses and meta-regression

Variable	Criteria
Primary effect measure of interest	Change in standardised mean difference effect size because of psychometrically informed re-analysis
Secondary effect measure of interest	Difference in the number and percentage achieving remission per trial
	Absolute risk reduction
Statistical analysis method	Random-effects analysis
Covariates	Age
	Gender Country Antidepressant type

### Ethics and consent

Ethical approval for this study was awarded by the RCSI University of Medicine and Health Sciences Ethics Committee (approval number 212560819). Consent to participate is not applicable to this protocol.

## Discussion

Although advanced psychometric analyses are recommended in methodological literature,^1–3^ it is unclear whether they matter to randomised trials in terms of their results on treatment efficacy. Our study will be the first to reanalyse IPD from antidepressant randomised trials to determine whether the use of confirmatory factor analysis techniques will modify the obtained effect sizes. A key strength and novel aspect of this study is that it will provide pooled psychometric models of depression pre- and post-treatment, according to the HRSD-17, which may inform better measurement of depression in the future. The results will have implications for clinical and social sciences more generally, in that, depending on the findings, they will highlight the relative worth of CFA modelling in intervention studies. Using factor scores for effect size analyses may eliminate measurement error, yielding larger effect sizes than currently reported. This would be theoretically and practically important, as it would demonstrate that advanced psychometric techniques are important for depression trials (and possibly all trials that use participant-reported outcomes) as well as increase clinician and patient confidence in antidepressant therapies. Conversely, if there are no discernible changes to the trial effect sizes, then the psychometric models may inform us which items are non-discriminatory in these trials, providing opportunities to reduce the numbers of items and measurement burden, with no negative connotations for trials or statistical power.

The study will have several limitations. Among these is the fact that the study is not re-addressing the actual effect sizes of antidepressant treatment, which, as outlined previously, have been established.^8,9^ By necessity, we will be adopting a different analysis plan from what would have been originally used in the individual trials. Therefore, any obtained effects previously published will not necessarily correspond to what we find. Although we will have the potential to achieve a large sample, a limitation of this study arising from time and resource constraints is that samples being used are specific to respective international clinical trial databases, which is compounded by the difficulties posed by adding other studies to the closed data environments operated by CSDR and Vivli.org, respectively. In addition, the results we obtain may not generalise to other psychometric modelling techniques, measures or indeed other therapies. It should also be noted that we will not attempt to statistically reconcile potential discrepancies in findings garnered from CSDR and Vivli.org data-sets, but rather will report these discrepancies and their potential implications. The potential for two differing, but statistically sound, models would not harm our primary effect size analyses, as we will be clarifying upon publication of findings that these findings are sample specific, and are predicated on depression being modelled, factor scores being derived and effect sizes being analysed from one respective sample. Future research will also examine the effects of confirmatory factor analysis in psychotherapy trials and the effects of other psychometric techniques, such as item response theory modelling and network analysis.^1–3^ Stakeholder opinions on how to interpret and implement the findings of the current and future work will also be ascertained.

## Supporting information

Doyle et al. supplementary material Doyle et al. supplementary material 1

Doyle et al. supplementary material Doyle et al. supplementary material 2

Doyle et al. supplementary material Doyle et al. supplementary material 3

Doyle et al. supplementary material Doyle et al. supplementary material 4

## Data Availability

Data availability is not applicable to this article as no new data were created or analysed in this protocol.
