# Supplementary material for: The effects of advanced factor analysis approaches on outcomes in randomised trials for depression: protocol for secondary analysis of individual participant data
Source: BJPsych Open. 2023 Aug 11;9(5):e157. doi: 10.1192/bjo.2023.544 (PMC10594098; doi:10.1192/bjo.2023.544)
Supplement: Doyle et al. supplementary material — 1 [file S2056472423005446sup001.docx]

**Supplementary Item 1: Statistical Analysis Plan**

Contents

[1. STUDY TITLE 2](#_Toc127382665)

[2. AIMS AND OBJECTIVES 2](#_Toc127382666)

[3. DATA SOURCES 2](#_Toc127382667)

[4. OUTCOMES 3](#_Toc127382668)

[4.1 Outcome assessment: Hamilton Rating Scale for Depression 3](#_Toc127382669)

[4.2 Psychometric modelling of depression 3](#_Toc127382670)

[4.3 Primary Outcome 3](#_Toc127382671)

[4.4 Secondary Outcome 4](#_Toc127382672)

[5. POPULATION 4](#_Toc127382673)

[6. STATISTICAL PROCEDURES 4](#_Toc127382674)

[6.1 Overview 5](#_Toc127382675)

[6.2 Psychometric Analyses 5](#_Toc127382676)

[6.2.1 Establishing psychometric models of depression at baseline (pre-treatment) and outcome (post-treatment) 5](#_Toc127382677)

[6.2.2 Comparing psychometric models of depression 8](#_Toc127382678)

[6.3 Modelling original and psychometrically informed data, and calculating effect sizes 9](#_Toc127382679)

[6.3.1 Computing weighted factor scores 9](#_Toc127382680)

[6.3.2 Primary Outcome: HRSD-17 scores 10](#_Toc127382681)

[6.3.3 Secondary Outcome: Proportions achieving remission 12](#_Toc127382682)

[6.4 Subgroup Analyses 14](#_Toc127382683)

[Exploring measurement invariance and potential gender effects 14](#_Toc127382684)

[References 15](#_Toc127382685)

# STUDY TITLE

The effects of advanced factor analysis approaches on depression randomised trial outcomes: Secondary analysis of individual participant data

# AIMS AND OBJECTIVES

By conducting secondary analyses of individual patient data (IPD) from randomised trials of antidepressant therapies, the overall aim of this study is to examine the effects, if any, of advanced psychometric analyses.

Specific objectives are:

1. To establish psychometric models of depression pre- (baseline) and post-treatment (outcome), using the Hamilton Rating Scale for Depression 17-item version (HRSD-17), yielding one or more factors of depression.
2. To determine the overall psychometrically-informed depression effect size according to treatment group and compare to the original overall treatment effect size.
3. To compare psychometrically-informed effect sizes to original effects across individual trials.
4. To compare proportions achieving remission using risk differences according to the recommended thresholds for the HRSD-17, versus the equivalents for the new psychometrically informed data across trials.

# DATA SOURCES

Data has been requested from two international databases; clinicalstudydatarequest.com (CSDR) and vivli.org (Vivli). Data obtained from CSDR and Vivli will be analysed separately. The planned analyses will be conducted using the Vivli dataset first, and then reiterated using the CSDR dataset to allow for the comparison of resulting models and effect size outcomes. Table S1 outlines the expected sample from each database.

Table S1. Expected Sample

| **Database One:** |  | **www.clinicalstudydatarequest.com (CSDR)** | |
| --- | --- | --- | --- |
| Number of studies requested | - | 65 | |
| Number of studies by sponsor  *GlaxoSmithKline* | - | 65 | |
| Approximate total sample | - | Circa 10,000 | |
|  |  |  | |
| **Database Two:** |  | **www.vivli.org (Vivli)** |  |
| Number of studies requested | - | 42 |  |
| Number of studies by sponsor  *Eli Lilly and Company*  *GlaxoSmithKline*  *Takeda*  *Pfizer* | -  -  -  - | 13  8  9  12 |  |
| Approximate total sample | - | Circa 10,000 |  |

# OUTCOMES

## 4.1 Outcome assessment: Hamilton Rating Scale for Depression

The Hamilton Rating Scale Depression (HRSD)^1^ is among the most widely used clinician-administered assessment instrument in randomised depression trials. Originally conceived as a 17-item composite scale, the HRSD is currently available in versions ranging from 7-31 items in length and has been published in several different languages. This study will garner IPD from the 17 items that are common to the most frequently used version in order to maximise sample size (HRSD-17). These 17 items are specifically designed to measure melancholic and physical symptoms of depression and vary in their scale of measurement, with seven items measured on a three-point scale (usually from 0-2) and 10 items measured on a five-point scale (usually from 0-4).

## 4.2 Psychometric modelling of depression

Psychometric modelling of depression will follow a process of exploratory and confirmatory analysis. Parallel analysis and exploratory factor analysis (EFA) will be used to examine the factorability, dimensionality and factor structure of a randomly split ‘exploratory dataset’. Factor solutions will be examined for baseline (EFA baseline) and outcome (EFA outcome). Confirmatory factor analysis (CFA) will then be conducted using a ‘confirmatory dataset’ to examine three models of the proposed EFA structure for both baseline and outcome:

1. CFA baseline – simple structure

CFA outcome – simple structure

1. CFA baseline – higher order structure

CFA outcome – higher order structure

1. CFA baseline – bi-factor structure

CFA outcome – bi-factor structure

While not a direct focus of this work, additional analyses will see these steps repeated separately for placebo and treatment arms at outcome to examine potential differences according to treatment received.

Weighted total factor scores will be computed from the optimum (CFA) model of depression at baseline and outcome (psychometrically-informed factor score data).

## 4.3 Primary Outcome

Multilevel linear regressions will be run to estimate potential effect sizes for the original raw data and the psychometrically-informed factor score data. The resulting effect sizes will be compared (original raw data vs psychometrically-informed factor score data) and any resulting difference will be examined as the effect size of interest. Additional models will also examine the influence of participant age and sex.

## 4.4 Secondary Outcome

The number and percentage of participants achieving remission, as well as differences in absolute risk reduction will be assessed. These analyses will be conducted using the recommended^1^ remission threshold of ≤ 7 (i.e. 13% of the total possible score) for the original data, and a psychometrically-determined threshold (i.e., 13% of the scale factor score – discussed further in section 6.3.3) for the weighted factor score data. Remission rates and absolute risk differences will then be examined, to identify potential differences in the number of participants achieving remission according to the original and psychometrically-informed data.

# POPULATION

This study will focus on individuals 18 years and older who have participated in phase II, III or IV randomised controlled antidepressant treatment trials. The sample characteristics of this study are outlined in Table S2.

Table S2. Sample Characteristics

| **Variable** |  | **Criteria** |
| --- | --- | --- |
| Phase | - | II, III and IV RCT |
| Population | - | Participants with any depressive disorder aged 18 years and older included in antidepressant treatment trials |
| Treatment | - | Antidepressant medication (any medication used in the context of treating depression) |
| Comparator | - | Any comparator (to establish baseline psychometric model); Placebo (to establish efficacy) |
| Timing of Outcome Assessment | - | 8 weeks (or within a band of 4-12 weeks), as per Cipriani et al.^2^ |
|  |  |  |
| Exclusion Criteria | - | All studies outside of the above parameters |

# STATISTICAL PROCEDURES

## 6.1 Overview

We will first examine three psychometric models of depression. The first will be a simple structure, which may be unidimensional or consist of multiple correlated or uncorrelated factors. We will also examine a higher-order model and a hierarchical bi-factor model, (Figure S1). These models will be examined pre- (baseline) and post-treatment (outcome) using the HRSD-17, to obtain one or more factors.

The factor structure of the optimum baseline model and optimum outcome (treatment and placebo combined) model of depression will then be used to compute total factor scores. Multilevel regression models incorporating a random effect for trial will be used to ascertain factor-informed effect sizes, which will in turn be compared to the effect sizes of the original raw trial data to assess statistical and clinically important differences in trial outcomes.

Subsequently, for each individual trial dataset, factor-informed effect sizes will be ascertained and compared to the effect sizes of the original raw trial data. Differences in individual trial effects (original and modified) will then be meta-analysed to obtain a pooled effect size of change between the original and modified data.

Each analysis will be conducted separately for CSDR and Vivli databases. Potential differences in analyses conducted in each database will be reported, in terms of both their psychometric properties (dimensionality, factor structure, fit indices etc.) and primary/secondary outcomes (differences in effect sizes and remission rates). These methods are described in more detail in the sections below.

## 6.2 Psychometric Analyses

### 6.2.1 Establishing psychometric models of depression at baseline (pre-treatment) and outcome (post-treatment)

A sequence of analyses will be conducted separately in each database (CSDR and Vivli). In line with the approach recommended by Lubke and Lunningham^3^, data will be randomly split into exploratory and confirmatory datasets, as per recommended procedures. We will undertake to perform this split in relation to each individual trial and within treatment group to ensure an equitable representation of each trial in the exploratory and confirmatory datasets. Parallel analysis with 1000 replications will be used in the exploratory sample to determine dimensionality. We will reject any factors that come after an acute inflection in the scree plot, along with eigenvalues lower than those that were randomly generated in the parallel analysis, as recommended by Hayton et al^4^. Then, using the *Psych* package in R^5^, exploratory factor analysis (EFA) – with an oblique factor rotation method to allow factors to correlate – will be used to examine the factorability and factor structure of the exploratory sample data^2^. EFA will be performed iteratively, removing items with factor loadings below 0.3 and/or communalities below 0.4, and adjusting the model structure until appropriate models are identified^6^.

If the results of parallel analysis and EFA are unclear or ambiguous, Mokken scaling procedures, using the Mokken package in R^7^, will then be used to examine further the most appropriately fitting model for the data. Mokken analysis will be conducted in line with the methods recommended by Meijer & Baneke^8^. This involves increasing the co-efficient value c in increments, commencing at c=.01, then in increments of .05 (c=.05, .10, .15, .2, .25, .3, .35, .4, .45, .5, .55, .6) until the most interpretable solution is found. This procedure excludes items that demonstrate poor discriminability, and the solution which best corresponds with the parallel analysis and EFA will be selected. As this may result in the exclusion of non-discriminating items, the final EFA solution may not include the full range of 17 HRSD items.

Confirmatory factor analyses (CFA), using the Lavaan package in R^8^, will then be conducted using the confirmatory sample dataset, examining the models found in the exploratory stage, along with other well-established models. Maximum likelihood (ML) will be used for the model estimation. Multivariate normality of the data will be examined using the Henze-Zirkler test^10^, which will determine whether a robust maximum likelihood method will be required when estimating model parameters. Model selection criteria will include Akaike Information Criterion (AIC) and Bayesian Information Criterion (BIC). Absolute fit indices will include Root Mean Square Error of Approximation (RMSEA) and Standardised Root Mean Square Residual (SRMR), which are considered acceptable at < 0.08^11^. Relative fit indices will include Comparative Fit Index (CFI) and Tucker-Lewis Index (TLI), which are considered acceptable at > 0.95^12^. Internal consistency will be assessed using McDonald’s Omega, which is typically considered acceptable at > 0.70^13^. Once EFA has determined the dimensionality and factor structure of the data, confirmatory factor analysis will be conducted in relation to the simple structure EFA model, along with two common factor analytical models that are well defined and replicated in psychopathology research: a higher-order model and a hierarchical bi-factor model*.*^1^ (see Figure S1). Chi-squared difference tests of these analytical models, together with examination of relevant fit indices, will be used to determine the optimum models of depression.


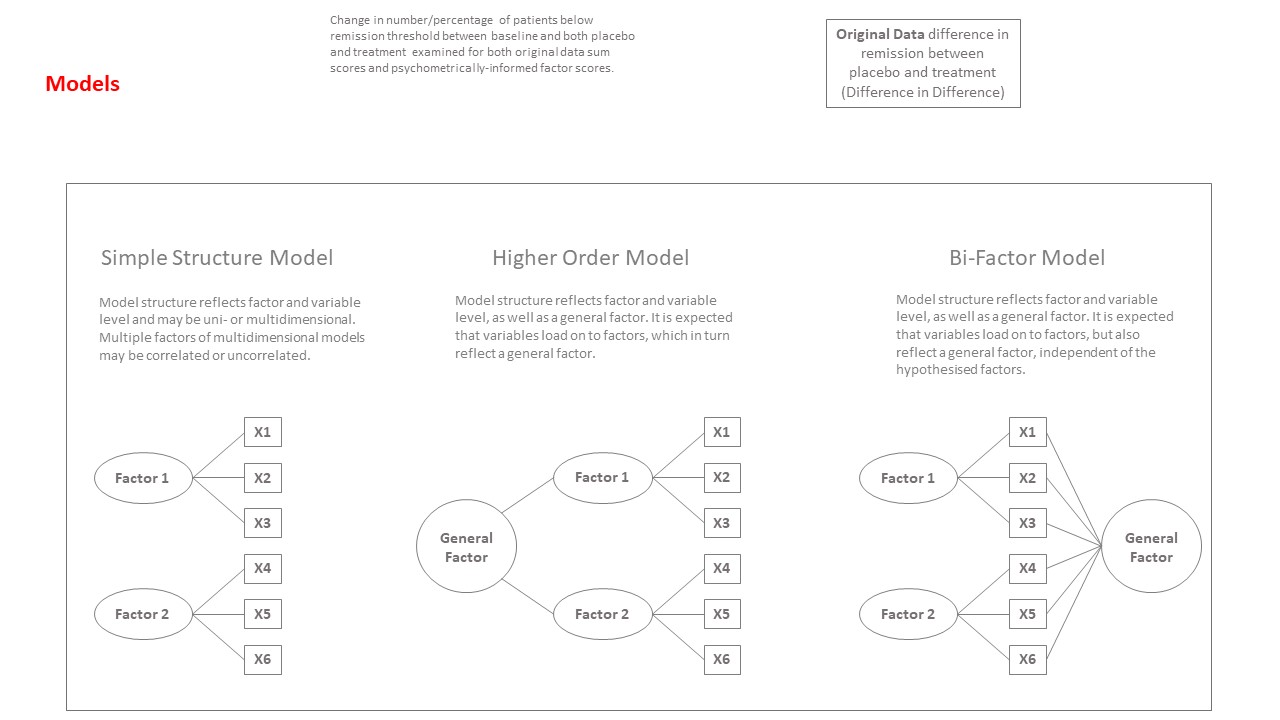


Figure S1. Illustrative Bi-Dimensional Example of a Simple Structure Model, a Higher Order Model and a Hierarchical Bi-Factor Model).

This analysis described above will run four times, (i) baseline data only, (ii) combined treatment and placebo outcome data at 8-weeks (4-12 weeks), (iii) outcome data in the treatment group only, (iv) outcome data using placebo group only, as outlined in Table S3. Results will be assessed across samples by comparing dimensionality and factor structure, as well as indicators such as factor loadings/cross loadings, communalities, variance explained.

Table S3. Exploratory and Confirmatory Factor Analysis Models to be Examined

|  | Model Name |  | Model Description |
| --- | --- | --- | --- |
|  | Exploratory Factor Analysis (using exploratory dataset) | | |
|  | EFA Baseline | - | EFA of baseline data |
|  | EFA Outcome | - | EFA of (combined) outcome data |
|  |  |  |  |
|  | EFA Placebo | - | EFA of (individual) outcome placebo data |
|  | EFA Treatment | - | EFA of (individual) outcome treatment data |
|  |  |  |  |
|  | Primary Models for Confirmatory Factor Analysis (using confirmatory dataset) | | |
| (i) | CFA Baseline - Simple | - | CFA of baseline data using simple structure identified by EFA |
|  | CFA Baseline - Higher Order | - | CFA of baseline data using higher order structure |
|  | CFA Baseline - Bi-factor | - | CFA of baseline data using bi-factor structure |
|  |  |  |  |
| (ii) | CFA Outcome - Simple | - | CFA of (combined) outcome data using simple structure identified by EFA |
|  | CFA Outcome - Higher Order | - | CFA of (combined) outcome using higher order structure |
|  | CFA Outcome - BI-factor | - | CFA of (combined) outcome using bi-factor structure |
|  |  |  |  |
|  | Additional Models for Confirmatory Factor Analysis (using confirmatory dataset) | | |
| (iii) | CFA Placebo - Simple | - | CFA of outcome placebo data using simple structure identified by EFA |
|  | CFA Placebo - Higher Order | - | CFA of outcome placebo data using higher order structure |
|  | CFA Placebo - BI-factor | - | CFA of outcome placebo data using bi-factor structure |
|  |  |  |  |
| (iv) | CFA Treatment - Simple | - | CFA of outcome treatment data using simple structure identified by EFA |
|  | CFA Treatment - Higher Order | - | CFA of outcome treatment data using higher order structure |
|  | CFA Treatment - BI-factor | - | CFA of outcome treatment data using bi-factor structure |

The optimum baseline model (or models, as the optimised solution may differ in the databases) and CFA outcome model will then be used to compute factor scores for each data-group, which will be used in later analyses (see Section 6.3). Factor scores will be computed using the *lavPredict* function in Lavaan, as per Farahdel et al^15^. Figure S2 provide an overview of the planned psychometric analyses. Note that only the 17 items of the HRSD which are common across all studies will be considered for this analysis (i.e. studies which use long version of the HRSD will have those items excluded from this analysis).


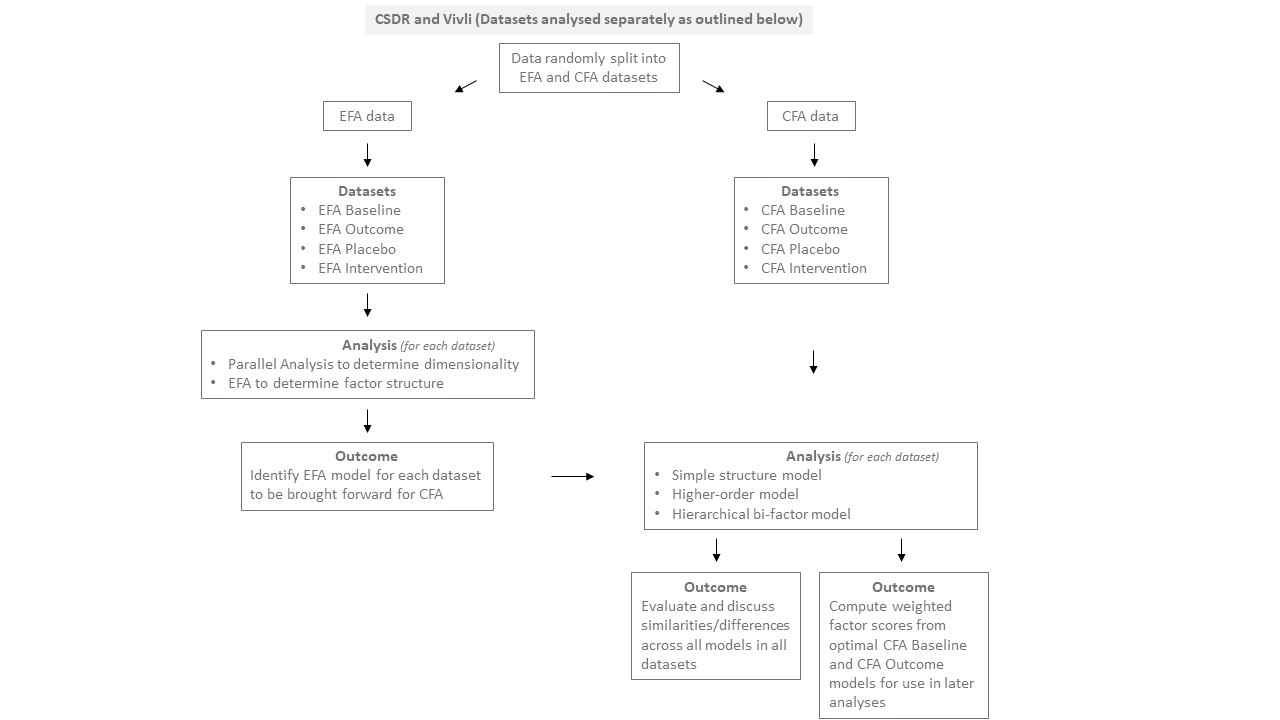


Figure S2. Overview of the planned psychometric analyses

### 6.2.2 Comparing psychometric models of depression

The models produced at baseline and outcome (placebo and treatment combined, as well as placebo and treatment separately) will be examined to identify any potential differences in model structure or item functioning. This will include examination of differences in nonperforming (i.e., dropped) items, examination of the factors onto which each item loads and inspection of the salience of each item according to their respective factor loading scores. EFA models that cannot be confirmed by CFA, as well as any potential differences between models produced using CSDR and Vivli data, will also be reported in detail. Respective analyses will continue as planned and potential differences in primary and secondary outcomes between CSDR and Vivli datasets will be examined and reported. Potential differences between baseline and/or outcome models and the original 17-item model proposed by Hamilton^1^ will also be assessed in terms of factor structure and item functioning/redundancy. Additional analyses will include sub-group analysis examining for potential gender invariance in all CFA models (see Section 6.4). The parameters of the psychometric analyses are outlined in Table S4.

Table S4. Parameters of Psychometric Analyses

|  |  | | **Criteria** | | |  |
| --- | --- | --- | --- | --- | --- | --- |
| Statistical Analysis Methods | | - | | Psychometric modelling of pre-treatment (baseline) and outcome data using exploratory and confirmatory factor analysis |  | |
| Rotation (EFA) | | - | | Oblique (e.g. Direct Oblimin) |  | |
| Estimator (CFA) | | - | | (robust) Maximum Likelihood |  | |
| Fit indices of Interest | | - | | Akaike Information Criterion (AIC) |  | |
|  |  | - | | Bayesian Information Criterion (BIC) |  | |
|  |  | - | | Root Mean Square Error of Approximation (RMSEA) | [< 0.08] | |
|  |  | - | | Standardised Root Mean Square Residual (SRMR) | [< 0.08] | |
|  |  | - | | Comparative Fit Index (CFI) | [> 0.95] | |
|  |  | - | | Tucker-Lewis Index (TLI) | [> 0.95] | |
| Internal Consistency | | - | | McDonald’s Omega | [> 0.70] | |
| Measurement of Differential Item Functioning | | - | | Communalities  Factor Loadings | [> 0.40]  [> 0.30] | |
|  |  | - | | r-squared |  | |
|  |  | - | | Mean |  | |
|  |  | - | | Standard Deviation |  | |
|  |  | - | | Standard Error |  | |
| Model Comparison | | -  - | | Fit Indices  Chi-squared difference test for factor analytical models | | |
| Planned Subgroup Analysis | | -  - | | Gender invariance using chi-squared difference tests  Change in fit indices  RMSEA  SRMR  CFI  TLI | [0.015]  [0.030]  [0.010]  [0.010] | |

## 6.3 Modelling original and psychometrically informed data, and calculating effect sizes

### 6.3.1 Computing weighted factor scores

Factor score weights will be garnered from the optimum models for baseline and combined outcome and used to obtain factor scores, which will be used in later analyses (see Section 6.3). These will be obtained using the *LavPredict* function in Lavaan^6^*.* Factor scores will differ from the sum scores used in the original analyses, as they will be weighted according to the variance explained by each item. Items that make a larger contribution to the variance explained will see participants’ scores increase, while items that make a smaller contribution will see scores decrease. LavPredict functions such that weighted scores can be derived for individual item responses for each participant. These can then be summed to calculate factor scores for each factor in the model, as well as a total scale factor score, the mean of which reflects the sum of the means of each individual factor^6^. To reflect current best practice for analysing randomised trials^17^, we will be conducting our analyses using the overall scale factor score. The way in which the weighted scores are derived results in the mean of participants’ weighted total factor scores being identical to their original raw scores. However, the redistribution of weight across items may result in a decrease in measurement error, which, in turn, could translate into a change in effect size. It should be noted that the number of items retained by each model may differ both from the original 17-item model of the HRSD and from each other. This will be accommodated in the analyses, as we will be comparing standardised mean differences as the effect of interest.

Ultimately, we will examine the effect size for placebo versus treatment using the raw total summed HRSD-17 scores, as per the original trial data. We will then compare this to the effect size for placebo versus treatment using the total summed factor scores derived from psychometric analyses using factor score weights garnered from the optimum models for baseline and combined outcome (See Table S3).

### 6.3.2 Primary Outcome: HRSD-17 scores

A sequence of analyses will be performed to establish whether there is any evidence that the application of factor scores modifies the obtained effect sizes. Primary (and secondary) outcome analyses will be conducted using the Vivli dataset, then reiterated using the CSDR dataset to identify potential differences in outcomes. First, we will establish the overall effects of antidepressants versus placebo in the pooled data, using original (raw score) HRSD-17 scores, by using a multilevel linear regression model, predicting outcome HRSD-17 scores, with treatment group as the predictor, adjusting for baseline HRSD-17 scores and study as the random intercept (Model 1). Then, a similar model will be built, except the total factor score at outcome (i.e. psychometrically-informed outcome sum scores) will be modelled, adjusting for total factor score at baseline (Model 2). We will also re-run these two models (i.e., Models 1 & 2), additionally adjusting for participant age and sex (Model 3 & 4 respectively).

Table S5. Multilevel Linear Regression Models for Primary Analyses

| Model Name |  | Model Description |
| --- | --- | --- |
| Model 1 | - | Original HRSD-17 scores modelled, with treatment group as the predictor, adjusting for baseline HRSD-17 scores and study as the random intercept. |
|  |  |  |
|  |  |  |
| Model 2 | - | Psychometrically-informed outcome sum scores (obtained using factor score weights garnered from the optimum models for baseline and combined outcome), with treatment group as the predictor, adjusting for baseline psychometrically-informed HRSD-17 scores and study as the random intercept. |
|  |  |  |
|  |  |  |
|  |  |  |
|  |  |  |
| Model 3 | - | Model 1, additionally adjusting for participant age and sex |
| Model 4 | - | Model 2, additionally adjusting for participant age and sex |

In each case, effects will be transformed into Cohen’s *d* standardised mean difference (SMD) to allow for effect size comparison. Potential differences between the raw score SMD difference and factor score SMD difference will be examined as the effect of interest (i.e. Model 1 vs Model 2, and Model 3 vs Model 4). Multilevel linear regression analyses will be conducted using the *lmer* function in the lme4 package in R^16^. An overview of the planned analysis is outlined in Figure S3.


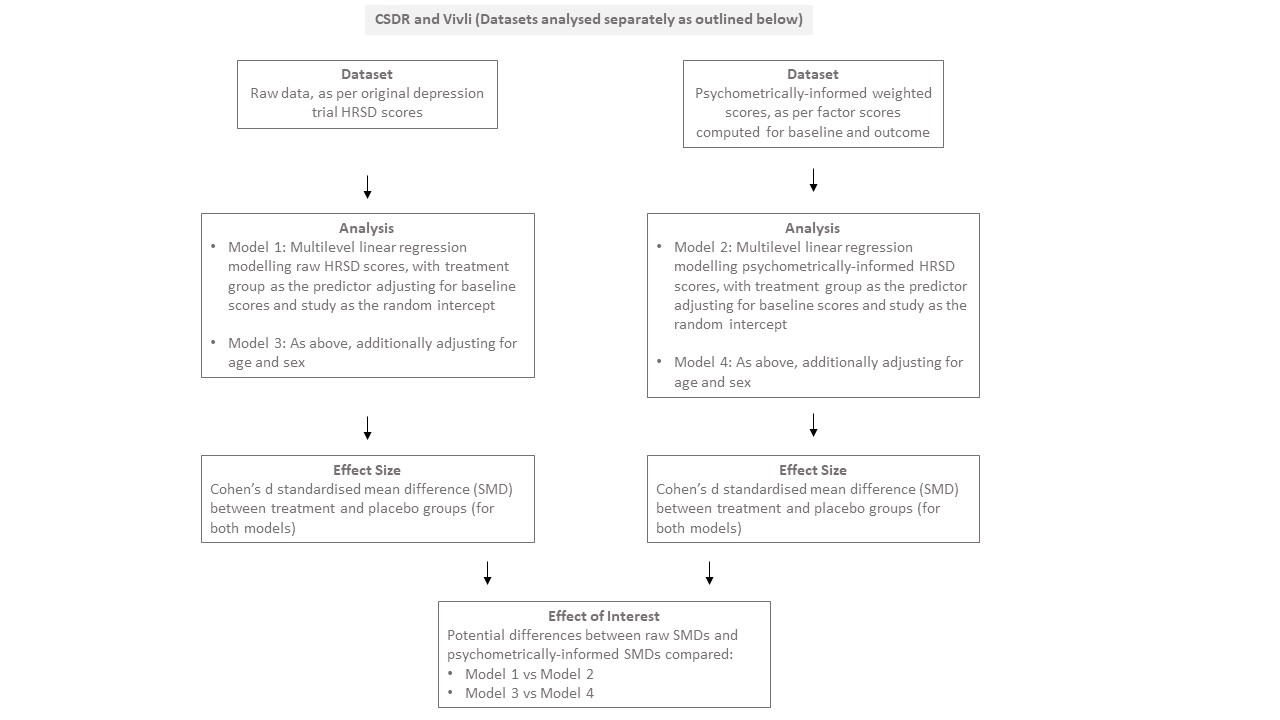


Figure S3. Overview of Analysis of Primary Outcome, Comparing Original Raw Data with Psychometrically-Informed Data

Furthermore, each trial will be examined individually, to assess changes per trial, and results combined in a meta-analysis. Linear regression analysis will be used separately for the original trial data and factor score data to assess potential changes in outcome scores adjusting for baseline scores. Linear regression analyses will be conducted using the *lm* function in R^16^. Effect sizes will be obtained for each trial, comparing the original effect sizes from the HRSD-17 total scores versus the factor-informed scores, again calculating SMDs. The difference between the (raw score) SMD and the factor-informed SMD is considered the effect of interest for each trial.

Differences in effect sizes from the original and factor score results will be calculated and examined using random-effects meta-analysis using the Stata command *meta^18^* to determine an overall change in effect size for the psychometrically-optimised data. Heterogeneity will be assessed using the I^2^ statistic, which is used to assess the percentage of variability in the effect estimates that can be attributed to heterogeneity rather than chance. We will also examine potential moderators, including study country, antidepressant type and gender, in a meta-regression analysis. The parameters of the multi-level regression are outlined in Table S6.

Table S6. Parameters of Multi-Level Regression

| **Variable** |  | **Criteria** |
| --- | --- | --- |
| Dependent Variable | - | HRSD-17 outcome sum scores |
| Predictor | - | Treatment group |
| Adjustment | - | HRSD-17 baseline sum scores |
| Random Intercept | - | Study |

### 6.3.3 Secondary Outcome: Proportions achieving remission

Clinically important differences between original and psychometrically-informed data will also be assessed by examining potential changes in the number and percentage of participants achieving remission, as well as differences in absolute risk reduction. These analyses will be conducted using the recommended^1^ remission threshold of ≤ 7. As psychometric analyses may result in the removal of non-performing items, it will be necessary to determine an equivalent threshold for use with the psychometrically-informed data. The current recommended threshold (≤ 7) as a percentage of the maximum possible depression score for the 17-item model (i.e. 52) is 13%. We will therefore calculate the revised threshold as 13% of the maximum possible score of the factor-informed model of the HRSD-17. For example, if analysis indicates a 12-item model with a maximum possible score of 39, 13% of this score would result in a depression threshold of ≤ 5 for the psychometrically informed model to achieve remission. Thus, 13% of the maximum score will be used to indicate remission for our secondary outcome analysis. Remission rates and absolute risk differences will then be examined, to identify potential differences in the number of participants achieving remission according to the original and psychometrically-informed data. Analyses will follow a three-step process:

1. The difference in number/percentage of patients below remission threshold between baseline and both placebo and treatment examined separately for both original data sum scores and psychometrically-informed factor scores.
2. Potential differences in remission for placebo versus treatment groups will be examined for both sum score and factor score data.
3. Potential remission difference in differences compared for sum scores versus factor scores which is the effect of interest.

Overall multilevel models will be built as per the primary analysis, this time using logistic regression analysing the participants achieving remission, using the raw data and the psychometrically-informed data. Further models will also adjust for age and sex, analogous to those described above. An overview of planned analysis can be seen in Figure S4.


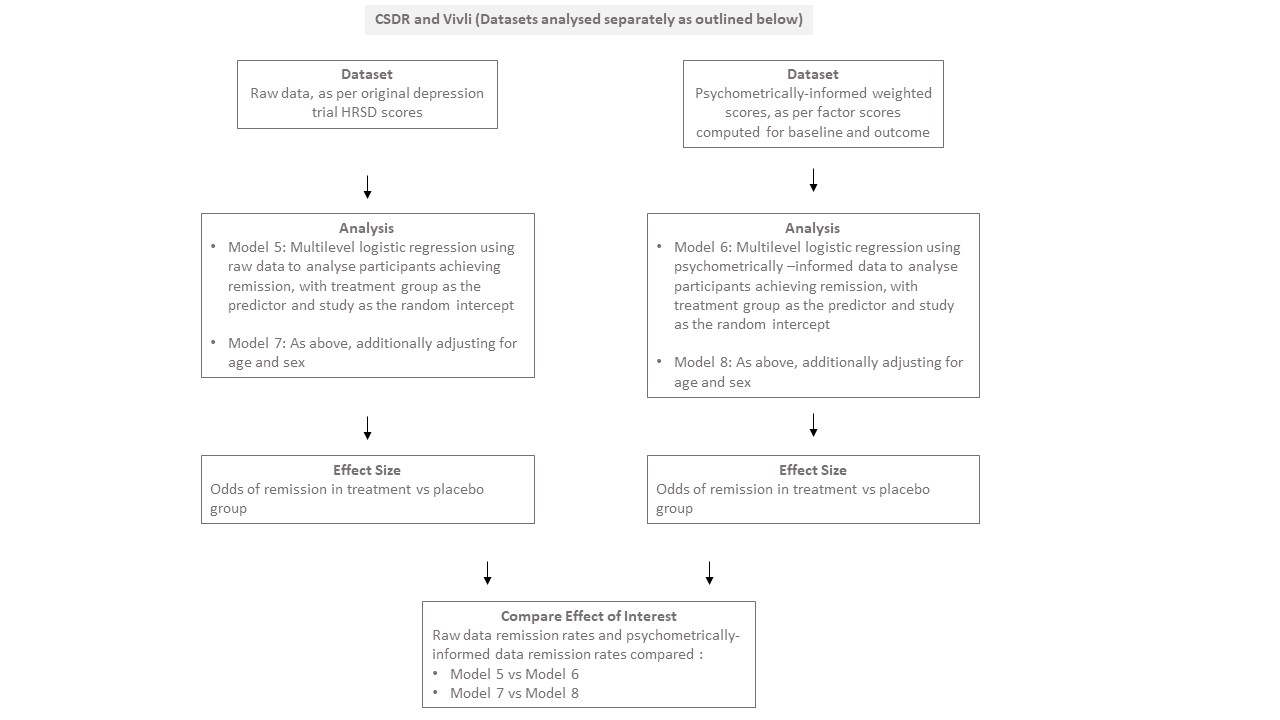


Figure S4. Overview of Analysis of Primary Outcome, Comparing Original Raw Data with Psychometrically-Informed Data

Then, we will use similar procedures to obtain the numbers achieving remission per trial, which will ultimately be modelled using random effects meta-analysis, with meta-regression exploring the potential moderators. The analyses will be conducted in Stata using the command *meta*^18^. The parameters for such are outlined in Table S7.

Table S7. Parameters of Meta-Analyses

| **Variable** |  | **Criteria** |
| --- | --- | --- |
| Primary Effect Measure of Interest | - | Change in SMD effect size due to psychometrically-informed re-analysis |
| Secondary Effect Measure of Interest | - | Difference in the number and percentage achieving remission per trial |
|  | - | Absolute Risk Reduction |
| Statistical Analysis Method | - | Random effects analysis with inverse variance |
| Covariates | - | Age |
|  | - | Gender  Country  Anti-depressant type |

## 6.4 Subgroup Analyses

### Exploring measurement invariance and potential gender effects

Potential gender effects will be assessed using measurement invariance methods recommended by Murray et al^19^ in the optimal CFA model. Configural invariance will be examined by identifying potential differences in model structure and non-conforming (dropped) items between the gendered models. Weak invariance will be examined by constraining factor loadings across gender groups, while strong invariance will be examined by constraining factor loadings and intercepts^9,19,20^. The presence or absence of gender invariance will be established by using the lavTestLRT function in Lavaan to examine for statistically significant differences in chi-squared values between^29^ the initial and constrained models. Invariance will also be determined in relation to fit indices using criteria recommended by Chen^21^, which advocates change thresholds of .010 for CFI, .015 for RMSEA and .030 for SRMR. TLI is not specifically mentioned by Chen, but will be assessed in relation to the same threshold as CFI (i.e. .010), as they share the same cut-off threshold^12^.

Differential item functioning will assessed by examining a range of statistics, such as factor loading scores, r-squared values, mean values, standard deviations and standard errors. If significant gender variance is found, individual psychometrically optimised models will be assessed using data for men and women separately^9,20^. Primary and secondary analyses will then be repeated using the gendered models.

# References

1. Hamilton M. The (21-item) Hamilton Rating Scale for Depression. 1967; Electronic source. Available at: http://healthnet.umassmed.edu/mhealth/HAMD.pdf.
2. Cipriani A, Furukawa TA, Salanti G, et al. Comparative efficacy and acceptability of 21 antidepressant drugs for the acute treatment of adults with major depressive disorder: a systematic review and network meta-analysis. *Lancet.* 2018; 391(10128):1357-1366.
3. Lubke GH, Lunningham J. “Fitting latent variable mixture models”. *Behavioural Research and Therapy.* 2017; 98(2): 91–102.
4. Hayton JC, Allen DG, Scarpello V. “Factor retention decisions in exploratory factor analysis: A tutorial on parallel analysis”. *Organizational Research Methods.* 2004; 7(2): 191–205.
5. Revelle W. "Procedures for Psychological, Psychometric, and Personality Research ". Psych. Last modified October 14, 2022. https://cran.r-project.org/web/packages/psych/psych.pdf.
6. Costello AB, Osborne J. “Best practices in exploratory factor analysis: four
   recommendations for getting the most from your analysis”. *Practice, Assessment, Research, and Evolution.* 2005; 10[Art 7]: 1-9.
7. Van Der Ark L, Koopman L, Straat HJ, Van Den Bergh D. "Conducts mokken scale analysis". Mokken. Last modified March 3, 2021. https://cran.r-project.org/web/packages/mokken/mokken.pdf
8. Meijer RR, Baneke JJ. Analyzing psychopathology items: a case for nonparametric item response theory modeling. *Psychological Methods.* 2004; 9: 354–368.
9. Rosseel Y, Jorgensen TD, Rockwood N, Oberski D, Byrnes J, Vanbrabant L, Savalei V, Merkle E, Hallquist M, Rhemtulla M, Katsikatsou M, Barendse M, Scharf F, Du H. "The lavaan tutorial". The Lavaan Project. Last modified May 2, 2022. https://lavaan.ugent.be/tutorial/tutorial.pdf.
10. Korkmaz S, Goksuluk D, Zararsiz G. "Multivariate normality tests". Multivariate Normality Tests. Last modified June 29, 2021. https://cran.r-project.org/web/packages/MVN/MVN.pdf
11. Schermelleh-Engel K, Moosbrugger M. "Evaluating the fit of structural equation models: Tests of significance and descriptive goodness-of-fit measures." *Methods of psychological research online.* 2003; 8(2): 23-74.
12. Smith TD, McMillan BF. "A Primer of Model Fit Indices in Structural Equation Modeling. Paper presented at the annual meeting of the Southwest Educational Research Association." Paper presented at Annual meeting of the Southwest Educational Research Association, New Orleans, 2001.
13. Hayes AF, Jacob JC. "Use Omega Rather than Cronbach’s Alpha for Estimating Reliability. But…." *Communication Methods and Measures.* 2020; 14(1): 1-24.
14. Burns A, Höfer S, Curry P, Sexton E, Doyle F. “Revisiting the dimensionality of the Hospital Anxiety and Depression Scale in an international sample of patients with ischaemic heart disease”. *Journal of Psychosomatic Research.* 2014; 77(2): 116–121.
15. Farahdel B, Thapaliya B, Suresh P, Ray B, Calhoun VD, Liu J. “Confirmatory Factor Analysis on Mental Health Status using ABCD Cohort”. IEEE *International Conference on Bioinformatics and Biomedicine (BIBM)*. 2021;: 3540-3547.
16. Bates D. Maechler M, Bolker B, Walker S, Christensen RHB, Singmann H, Dai B, Scheipl F, Grothendieck G. "Linear Mixed-Effects Models using 'Eigen' and S4". Lme4. Last modified November 1, 2022. https://cran.r-project.org/web/packages/lme4/lme4.pdf.
17. European Medicines Agency. “Guidelines on Adjustment for Baseline Covariates in Clinical Trials”. February 9, 2023. https://www.ema.europa.eu/en/documents/scientific-guideline/guideline-adjustment-baseline-covariates-clinical-trials_en.pdf
18. Sterne JAC. *Meta-Analysis in Stata: An Updated Collection from the Stata Journal.* College Station, TX: Stata Press; 2009.
19. Murray AL, Hemady CL, Do H, Dunne M, Foley S…Walker S. " Measuring Antenatal Depressive Symptoms Across the World:
    A Validation and Cross-Country Invariance Analysis of the Patient Health
    Questionnaire-9 (PHQ-9) in Eight Diverse Low-Resource Settings.”Psychological Assessment”*.* 2022; 34(11): 993-1007.
20. Quilty LC, Robinson JJ, Rolland JP, Fruyt FD, Rouillon F, Bagby R. M. "The structure of the Montgomery-Åsberg depression rating scale over the course of treatment for depression." *International Journal of Methods in Psychiatric Research.* 2013; 22(3): 175-184.
21. Chen FF. “Sensitivity of Goodness of Fit Indexes to Lack of Measurement Invariance”. Structural Equation Modelling: A Multidisciplinary Journal. 2007; 14(3): 464-504.
