# Supplementary figures and images for: The effects of advanced factor analysis approaches on outcomes in randomised trials for depression: protocol for secondary analysis of individual participant data
Source: BJPsych Open. 2023 Aug 11;9(5):e157. doi: 10.1192/bjo.2023.544 (PMC10594098; doi:10.1192/bjo.2023.544)

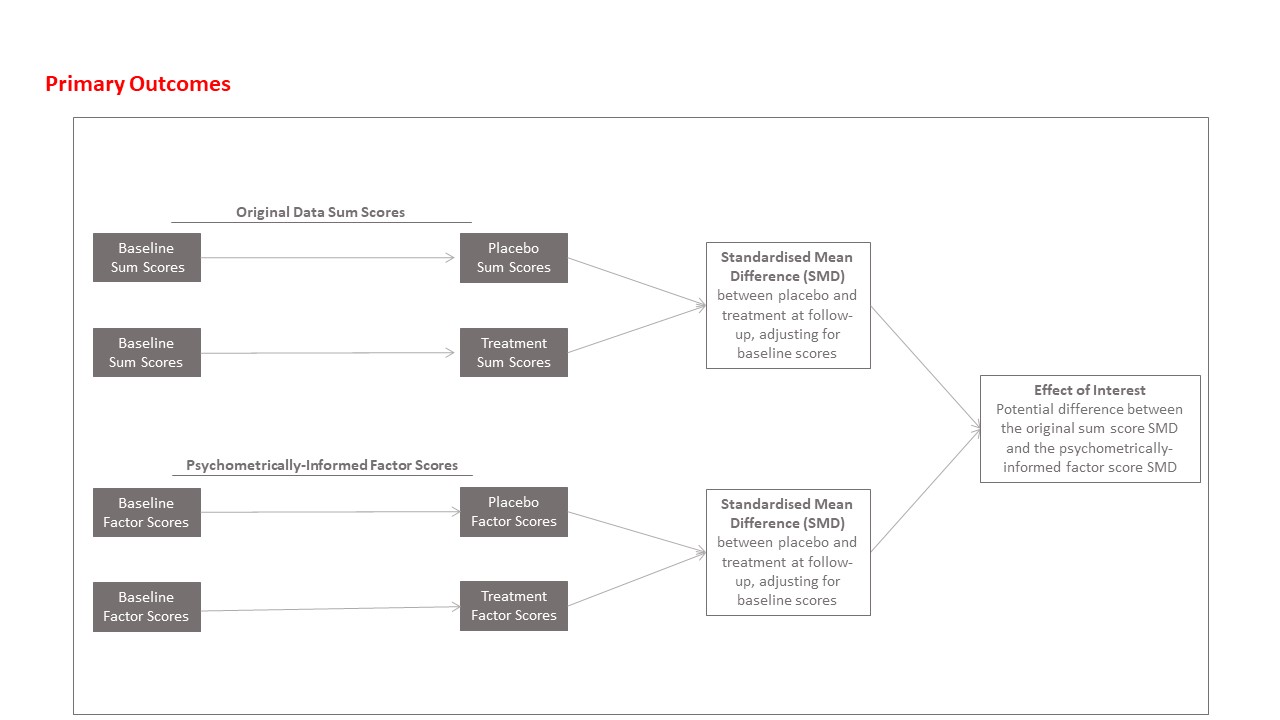

Supplement: Doyle et al. supplementary material — 2 [file S2056472423005446sup002.jpg]

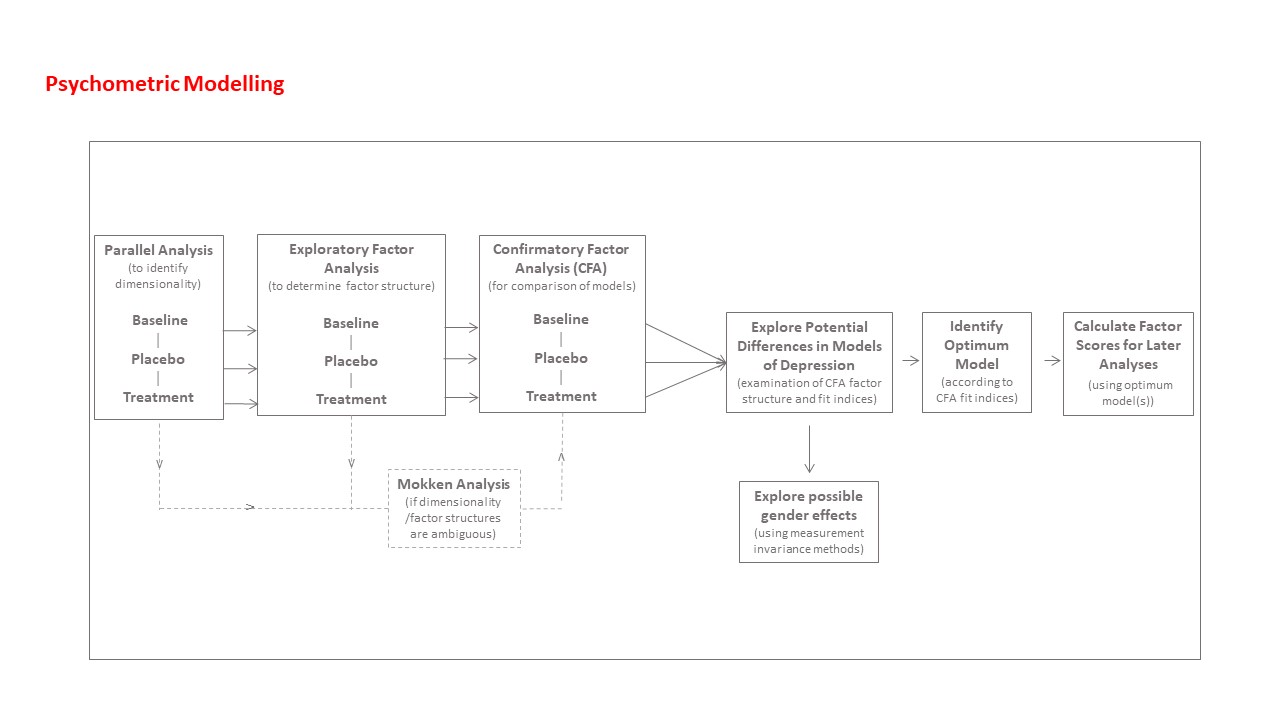

Supplement: Doyle et al. supplementary material — 3 [file S2056472423005446sup003.jpg]

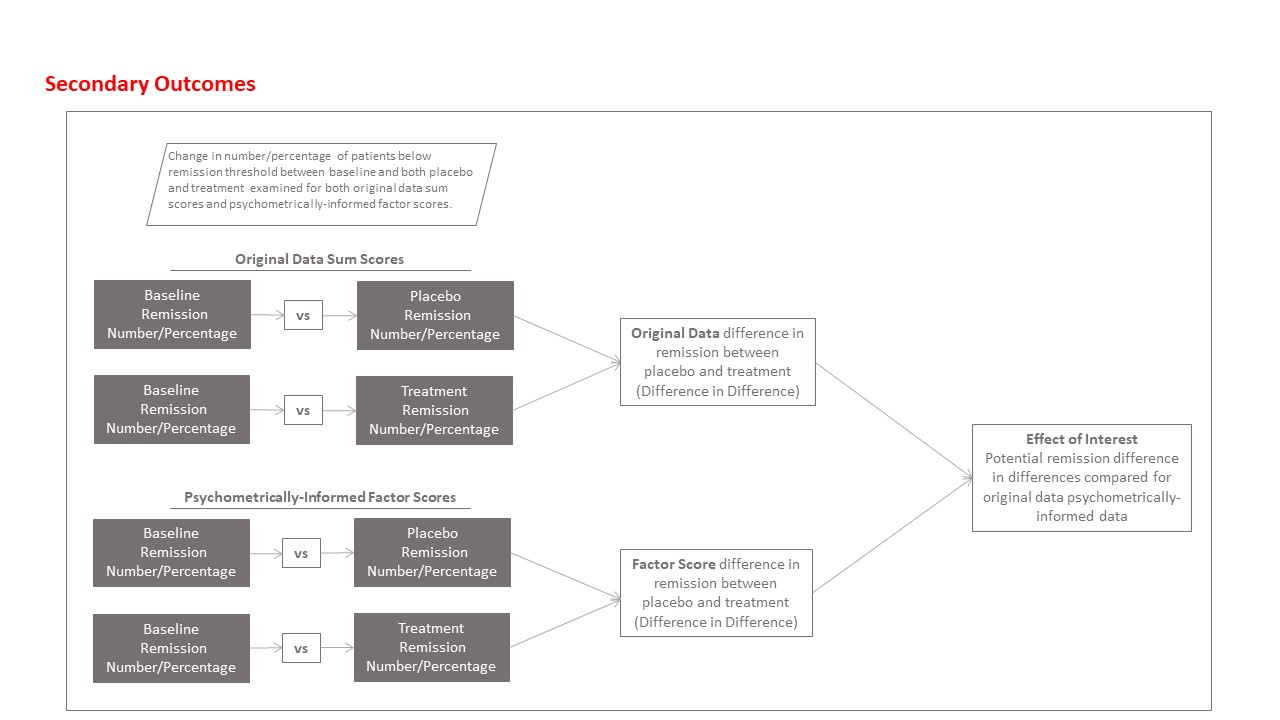

Supplement: Doyle et al. supplementary material — 4 [file S2056472423005446sup004.jpg]
